# Qualitative and quantitative detectability of hypertrophic olivary degeneration in T2, FLAIR, PD, and DTI: A prospective MRI study

**DOI:** 10.3389/fneur.2022.950191

**Published:** 2022-08-03

**Authors:** Eike Steidl, Maximilian Rauch, Elke Hattingen, Stella Breuer, Jan Rüdiger Schüre, Marike Grapengeter, Manoj Shrestha, Christian Foerch, Martin A. Schaller-Paule

**Affiliations:** ^1^Institute of Neuroradiology, University Hospital Frankfurt, Frankfurt am Main, Germany; ^2^Brain Imaging Center (BIC), Goethe-University Frankfurt, Frankfurt am Main, Germany; ^3^Department of Neurology, University Hospital Frankfurt, Goethe-University, Frankfurt am Main, Germany

**Keywords:** inferior olivary nucleus, stroke, diffusion MRI, proton density, connectivity, HOD, palatal tremor, cerebellum

## Abstract

**Purpose:**

Hypertrophic olivary degeneration (HOD) is a pathology of the inferior olivary nucleus (ION) that occurs after injuries to the Guillain-Mollaret triangle (GMT). Lacking a diagnostic gold standard, diagnosis is usually based on T2 or FLAIR imaging and expert rating. To facilitate precise HOD diagnosis in future studies, we assessed the reliability of this rater-based approach and explored alternative, quantitative analysis.

**Methods:**

Patients who had suffered strokes in the GMT and a matched control group prospectively underwent an MRI examination including T2, FLAIR, and proton density (PD). Diffusion tensor imaging (DTI) was additionally performed in the patient group. The presence of HOD was assessed on FLAIR, T2, and PD separately by 3 blinded reviewers. Employing an easily reproducible segmentation approach, relative differences in intensity, fractional anisotropy (FA), and mean diffusivity (MD) between both IONs were calculated.

**Results:**

In total, 15 patients were included in this study. The interrater reliability was best for FLAIR, followed by T2 and PD (Fleiss κ = 0.87 / 0.77 / 0.65). The 3 raters diagnosed HOD in 38–46% (FLAIR), 40–47% (T2), and 53–67% (PD) of patients. False-positive findings in the control group were less frequent in T2 than in PD and FLAIR (2.2% / 8.9% / 6.7%). In 53% of patients, the intensity difference between both IONs on PD was significantly increased in comparison with the control group. These patients also showed significantly decreased FA and increased MD.

**Conclusion:**

While the rater-based approach yielded the best performance on T2 imaging, a quantitative, more sensitive HOD diagnosis based on ION intensities in PD and DTI imaging seems possible.

## Introduction

Hypertrophic olivary degeneration (HOD) is a distinct pathology of the inferior olivary nucleus (ION). The ION is part of an anatomical-functional brain stem loop, the so-called dentato-rubro-olivary pathway that is also named Guillain-Mollaret triangle (GMT) in reference to its first describers (1). Within this inhibitory, unidirectional loop fibers from the dentate nucleus (DN) ascend through the superior cerebellar peduncle (SCP) to the contralateral red nucleus (RN) and then descend from the RN *via* the central tegmental tract (CTT) to the ipsilateral ION. The triangle is completed by fibers projecting from the ION to the contralateral DN *via* the inferior cerebellar peduncle and possibly the cerebellar cortex (2–6). As a distinctiveness of the GMT, lesions injuring the nuclei (DN, RN) or afferent fiber tracts (SCP, CTT) can cause deafferentiation and subsequent degeneration of the ION (2, 7, 8). This transsynaptic degenerative process has been characterized neuropathologically by Goto et al. and others (9–15), and its 6 stages can partially be depicted on MRI (5).

*Stages (1) and (2)*: No changes within the first 24 h after suffering the causal lesion, followed by degeneration of fibers around the ION; not visible on MRI.

*Stage (3)*: Hypertrophy of neurons and neurites starting at around 3 weeks; T2 hyperintensity of the ION.

*Stages (4) and (5)*: Additional hypertrophy of astrocytes starting at approximately 6 months, neurons begin to dissolute at approximately 9 months while gemistocytic astrocytes remain present; T2 hyperintensity and enlargement of the ION.

*Stage (6)*: Final stage with neuronal disappearance at around 3–4 years; lasting T2 hyperintensity and atrophy of the ION.

Besides the morphological appearance of HOD, the disinhibition of the ION is linked to a clinical syndrome consisting of a palatal tremor, vertical pendular nystagmus, and Holmes tremor of the upper limbs (16). Less frequently, cerebellar speech disorders or ataxia has been reported (17). Specifically in children, there is also a correlation with the posterior fossa syndrome, manifesting as cerebellar mutism subsequent to the resection of a cerebellar tumor (7, 18). Yet, HOD can also appear without clinical symptoms (19), and the exact underlying mechanisms remain unclear. Even data on the epidemiology and treatment options of HOD is scarce (16). Also, the vast majority of studies concerning the topic are retrospective, with very few exceptions (20–22). Furthermore, no study to date has recruited patients prior to the imaging diagnosis of HOD, which carries the risk of a sampling bias by analyzing mostly severe cases more likely to be detected incidentally in the clinical routine. Conducting prospective studies is challenging due to the rarity of the entity and the lack of a diagnostic gold standard for HOD. In previous studies, the diagnosis of HOD is usually based on T2 and/or fluid-attenuated inversion recovery (FLAIR) hyperintensity of the ION identified by one or more radiologists (5, 23–25). While HOD can easily be identified if a distinct hypertrophy is present, the mere T2 hyperintensity can be diffuse and subtle (2, 18, 26). Furthermore, most studies were retrospective studies and were not optimized for imaging of the brain stem, and the readers were not blinded to the patients' symptoms or causative lesions. Therefore, the reliability and reproducibility of this diagnostic approach are unknown. Apart from FLAIR and T2-weighted (w) imaging, proton density-weighted (PD-w) sequences have been discussed to be more sensitive to detect brain stem lesions (27, 28) but were never assessed in the context of HOD. Other studies assessed diffusion-weighted methods such as diffusion tensor imaging (DTI) in HOD patients. As the main finding, one study found a non–significant decrease in fractional anisotropy (FA) and a significant increase in diffusivity of the ION in HOD patients compared with controls (21), which is in line with previous case reports (29–31).

To support future studies in the field, we conducted this prospective analysis. Our objective was to assess the reliability of the commonly employed T2-w/FLAIR sequences and the rater-based approach for HOD diagnosis, analyze the possible benefit of additional PD-w brain stem imaging, and explore reproducible quantitative approaches for the diagnosis of HOD.

## Materials and methods

### Patient cohort

The study was conducted together with an ongoing multicenter study on the incidence and clinical features of HOD after stroke lesions in the GMT (German Clinical Trials Register ID: DRKS00020549, the trial protocol has previously been published (22)). We prospectively enrolled patients who met the following inclusion criteria:

(i) Stroke with topo-anatomical relation to the GMT, confirmed by MRI
(ii) Sufficient clinical condition for an additional MRI examination
(iii) At least 18 years old at initial diagnosis
(iv) Written and informed consent could be obtained.

Enrolled patients were examined with a dedicated MR protocol. The examinations were conducted at a minimum of 3 months after the initial stroke event. Further clinical data concerning, for example, the incidence and dynamic of the patients' symptoms will be published separately in the final analysis of the ongoing clinical trial.

In addition, an age- and sex-matched control group of healthy subjects was recruited for the current imaging study.

#### Imaging protocol

The examinations were conducted on 3 Tesla MRI scanners (Prisma® or Skyra® Siemens Healthineers, Erlangen, Germany) with an identical MRI protocol. The MRI protocol consisted of a high-resolution, dual echo PD/T2-w sequence optimized for imaging of the brain stem (time-to-echo: 12 ms/96 ms; time-to-repeat: 4,500 ms; flip-angle: 130°; slices: 25; slice thickness: 2 mm; field of view: 240 × 240 mm^2^; matrix size: 384 × 384); a 3D T1-weighted sequence for anatomical reference [magnetization prepared rapid acquisition with gradient echoes (MPRAGE); time-to-echo: 2.52 ms; time-to-repeat: 1,900 ms; flip-angle: 9°; slices: 192; slice thickness: 1 mm; field of view: 256 × 256 mm^2^; matrix size: 256 × 256]; a DTI sequence using HASE-EPI (32) (highly asymmetric spin echo echoplanar imaging; time-to-echo: 47 ms/11 ms; time-to-repeat: 7,200 ms; axial slices: 72; slice thickness: 2 mm without gap; field of view: 256 × 256 mm^2^; matrix size: 128 × 128; partial Fourier: 6/8; 2-fold acceleration; isotropic resolution: 2 mm; b-value: 1,000 s/mm^2^; 60 directions); and a FLAIR sequence that is also used in the clinical routine at our institution (time-to-echo: 81 ms; time-to-repeat: 8,500 ms; flip-angle: 150°; slices: 35; slice thickness: 4 mm; field of view: 220 × 220 mm^2^; matrix size: 320 × 320). Disease control subjects were examined with a short version of the protocol including only the FLAIR and double echo PD/T2-w sequences.

#### Data analysis

We performed two separate analyses, namely, a rater-based analysis and a quantitative analysis. For processing the MRI data, the FMRIB's Software Library (FSL, version 5.0.10, https://fsl.fmrib.ox.ac.uk/fsl) toolbox was used to analyze DTI data, and the software ITK-SNAP (version 3.6.0, www.itksnap.org, (33)) was used for segmentation.

The DTI datasets were analyzed using the FMRIB's diffusion toolbox, which is part of the FSL, version 5.0.10. Datasets were corrected for head motion, susceptibility, and eddy-current-induced distortions (*via* EDDY). Geometrical distortions caused by B0 field inhomogeneities were corrected *via* TOPUP based on additional inverted phase-blip data. For this purpose, five pairs of reference volumes at b = 0 were acquired with positive and negative phase-encoding gradients. Consecutively, non-brain tissue was removed from the averaged undistorted reference images using the FMRIB brain extraction tool (BET). Final preprocessing steps for eddy-current correction were performed *via* EDDY, as described in the literature (34, 35). FA and MD maps were computed by voxel-wise diffusion tensor fitting using linear regression, as implemented in the FSL toolbox (DTIFIT). Additional information on the DTI analysis has previously been published (22).

**Figure 1:**
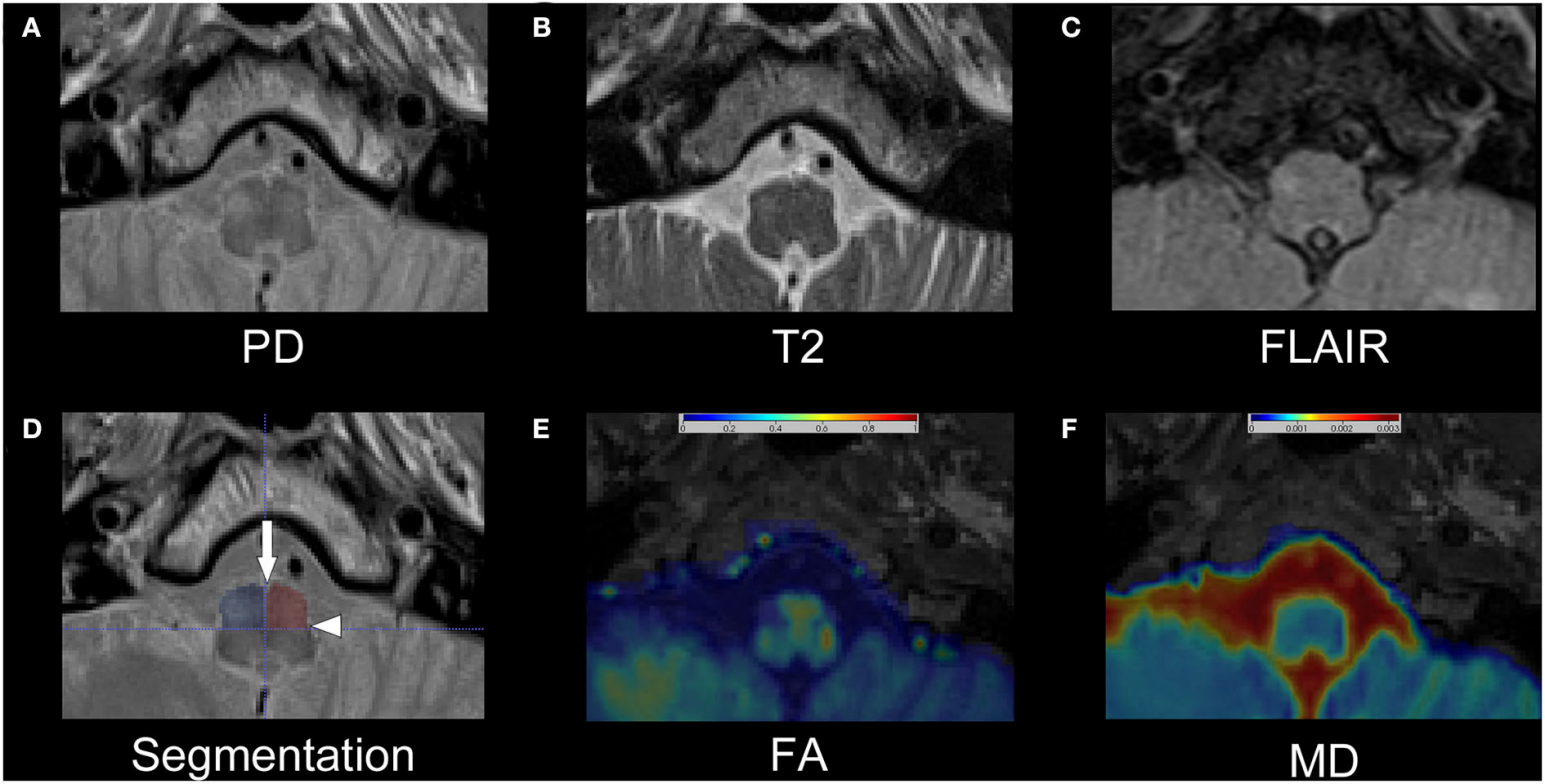
Exemplary dataset of patient number 13. The upper row shows the cropped PD-w **(A)**, T2-w **(B)**, and FLAIR **(C)** images that were used for the rater-based analysis. The segmentation of the anterior quadrants of the medulla oblongata is shown as semitransparent overlay on the PD-w image in **(D)** with an additional indication of the anterior median fissure (arrow) and the posterolateral sulcus (arrowhead). While the left ION is hardly visible, especially in the T2-w image and FLAIR, the right ION is hyperintense and possibly enlarged, indicating HOD. Images **(E)** and **(F)** show the corresponding FA and MD maps as interpolated and color-coded, semitransparent overlay on a T1-w image. In comparison with the left ION, a decrease in FA and an increase in MD are recognizable in the right ION. PD, proton-density; w, weighted; FLAIR, fluid-attenuated inversion recovery; ION, inferior olivary nucleus; HOD, hypertrophic olivary degeneration; FA, fractional anisotropy; MD, mean diffusivity.

For the reviewer-based analysis, we generated a “blinded” test dataset, so reviewers would not be influenced by the visible presence or absence of lesions within the GMT. Therefore, all individual PD, T2-w, and FLAIR datasets were cropped in all three dimensions to include only the medulla oblongata with the ION and to exclude the other structures of the GMT in the cerebellum and mesencephalon. An example of the cropped images is shown in Figures 1A–C. The individual, cropped PD-w, T2-w, and FLAIR datasets of the patients and disease controls were then sorted in random order. Three expert reviewers, all of whom with more than 6 years of experience in the field of neuroradiology, rated the test dataset. The reviewers were asked to identify the presence and laterality of a HOD separately in every individual PD-w, T2-w, and FLAIR dataset. The ratio of patients to disease controls within the test dataset was not disclosed.

Four additional PD-w, T2-w, and FLAIR datasets derived from additional disease controls were used as a training set to allow the reviewers to adapt to the cropped images and the visual appearance of the PD-w sequence (refer to Supplementary Figure 1 for examples).

For the quantitative analysis, a segmentation of the medulla oblongata with a focus on accessibility and reproducibility was established. Using the anterior median fissure and the posterolateral sulcus as easily identifiable anatomical landmarks, the medulla oblongata can be divided into quadrants (Figure 1D). Strictly following this approach, the medulla oblongata was segmented into quadrants on four consecutive slices on the two sequences with 2 mm slice thickness (T2/PD-w, DTI) and on two corresponding slices on the FLAIR sequences with 4 mm slice thickness. Thus, an 8-mm section of the medulla oblongata was segmented on all sequences. As the anatomical information of the FA and MD maps is limited through the limited in-plane resolution (2 × 2 mm^2^), the co-registered T1 MPRAGE datasets were used for additional anatomical reference. Based on these regions of interest (ROI), we computed the average intensities within the anterior quadrants of the medulla oblongata containing the ION. To normalize the results, the percentage differences between the mean intensities of the two quadrants were calculated for PD-w, T2-w and FLAIR datasets (*mean intensity a* [*side with higher mean*]−*mean intensity* *b*[*side with lower mean*]/*mean intensity b* [*side with lower mean*]). As stated in the formula, the ROI with the lower intensity was used as reference, resulting in positive percentage differences. Based on the distribution of values of these differences in the control cohort, a threshold containing 99% of all expectable values was calculated for each of the three sequences (*threshold* = *distribution mean* + 2.576^*^standard deviation). In the patient dataset, intensity differences exceeding this threshold were considered indicative of HOD. As there was no option for a comparison to the control cohort, the percentage differences for the FA and MD maps were calculated slightly different, considering the individual side that a HOD could be anticipated based on the causal index lesion as prior knowledge (*mean intensity a* [ *side with expected HOD*]−*mean intensity b* [*contralateral side*]/*mean intensity a*[*side with expected HOD*]). This resulted in both positive and negative percentage differences, allowing for an easier graphical interpretation. The calculated MD and FA differences were then compared between patient subgroups whose evaluation of the PD-w datasets was or was not indicative of HOD.

#### Statistics

Statistical testing was done employing GraphPad Prism (Version 9.3.1, GraphPad Software LLC, San Diego, United States). The interrater reliability was analyzed using the Fleiss' kappa. We used the Kolmogorov-Smirnov-Lilliefors test to test for normal distributions. If the data were normally distributed, we compared different measurements within the same group with the paired *t*-test and intergroup differences with the Welch test. For data without normal distribution, intergroup differences were tested with the Wilcoxon-Mann-Whitney test. We indicated all used tests in the “Results” section. A *p*-value of < 0.05 was deemed to indicate significance.

## Results

### Patients

We recruited 15 patients and 19 disease control subjects. The datasets of 4 control subjects were used as a training set for the reviewers and not included in the analysis as explained in the “Materials and methods” section.

There were no significant differences in age and sex distribution between the patient and control cohort (median age 59 vs. 63 years; range 33–80 and 49–79 years; *p* = 0.71/female patients 33% vs. 27%; *p* = 0.77; Wilcoxon-Mann-Whitney test). In the patient cohort, 10 patients had suffered ischemic strokes and 5 patients had suffered intracerebral hemorrhages. Ensuing lesions were most frequently located in the CTT (46.7 %). The median interval from the clinical event to the MRI examination was 4.4 months (3–59 months). Detailed patient characteristics are shown in Table 1.

**Table 1:** Patient characteristics.

**General characteristics**	
Total patient number; *n*	15
Age at diagnosis; years	
*Median (range)*	59 (33–80)
Sex; *n*	
*Female*	5 (33.3 %)
**GMT Lesions**	
Type; *n*	
*Ischemic*	10 (66.6 %)
*Hemorrhagic*	5 (33.3 %)
Location; *n*	
*CTT*	7 (46.7 %)
*DN*	6 (40 %)
*RN*	1 (6.7 %)
*SCP*	1 (6.7 %)
Interval clinical event to MRI; month
*Median (range)*	4.4 (3–59)

*GMT, Guillain-Mollaret triangle; CTT, central tegmental tract; DN, dentate nucleus; RN, red nucleus; SCP, superior cerebellar peduncle*.

PD/T2-w datasets were available in all patients and disease controls. FLAIR data were only available in 13 out of 15 patients (due to technical issues and severe motion artifacts, respectively) and in all 19 disease controls. DTI datasets were available in 14 patients and not acquired in disease controls.

### Rater-based analysis

All results are summarized in Table 2. Lacking a gold standard for the diagnosis of HOD, a classic calculation of sensitivity and specificity was not feasible. First, we evaluated the interrater reliability within the patient cohort. The interrater reliability was best for the FLAIR datasets (κ = 0.87) followed by the T2-w (κ = 0.77) and PD-w datasets (κ = 0.65). As a surrogate for the sensitivity, we analyzed the percentage of HOD diagnosis for the three reviewers individually in the patient datasets. The highest percentage of HOD diagnosis was made in the PD-w datasets (mean 60%; range 53.3–66.7%), with a notable difference in the T2-w and FLAIR datasets (mean 44.4 and 43.6%, respectively; range 40–46.7 and 38.5–46.2%, respectively). As the lower interrater reliability within the PD-w datasets led to an increased number of divergent ratings, the difference in the percentage of HOD diagnosis was less pronounced if a consensual diagnosis of all three reviewers was required (HOD diagnosis on PD-w: 40%; T2-w: 33.3%; FLAIR: 38.5%).

**Table 2:** Rater-based analysis.

	**Patient cohort**	**Control cohort**
**Interrater reliability;** *Fleiss' k*		
*PD*	0.65
*T2*	0.77
*FLAIR*	0.87	
**HOD diagnosis by**		
**individual reviewers**; %; mean *(range)*		
*PD*	60 *(53.3–66.7)*	8.9 *(0–13.3)*
*T2*	44.4 *(40–46.7)*	2.2 *(0–6.7)*
*FLAIR*	43.6 *(38.5–46.2)*	6.7 *(0–13.3)*
**thereof considered “false positive”**		
*PD*	8.9 *(6.7–13.3)*	8.9 *(0–13.3)*
*T2*	0	2.2 *(0–6.7)*
*FLAIR*	2.6 *(0-7.7)*	6.7 *(0–13.3)*
**HOD diagnosis in consensus**; %		
*PD*	40	0
*T2*	33.3	0
*FLAIR*	38.5	0

*PD, proton density; FLAIR, fluid-attenuated inversion recovery; HOD, hypertrophic olivary degeneration*.

To assess the specificity of the three sequences, we evaluated the percentage of false-positive findings for the three reviewers individually in the control group datasets. While there was a mean of only 2.2% false-positive HOD diagnosis in the T2-w datasets (corresponding to a single false-positive finding in all ratings), there was a mean of 6.7 and 8.9% false-positive diagnosis in the FLAIR and PD-w datasets, respectively. Since the laterality of the HOD was noted by the reviewers, it was also possible to identify false-positive findings in the patient cohort if the side of the HOD diagnosis does not correspond to the expected side considering the causative index lesion. While none of these false-positive diagnoses were found in the T2-w datasets, a mean of 2.6% false-positive findings appeared in the FLAIR datasets and a mean of 8.9% false-positive findings appeared in the PD-w datasets.

### Quantitative analysis

The segmentation ROIs in the left and right anterior medulla oblongata of the patient cohort had a mean volume of 554 mm^3^, containing a mean of 567 individual voxels in PD/T2-w and a mean of 39 individual voxels in the DTI-based datasets.

**Figure 2:**
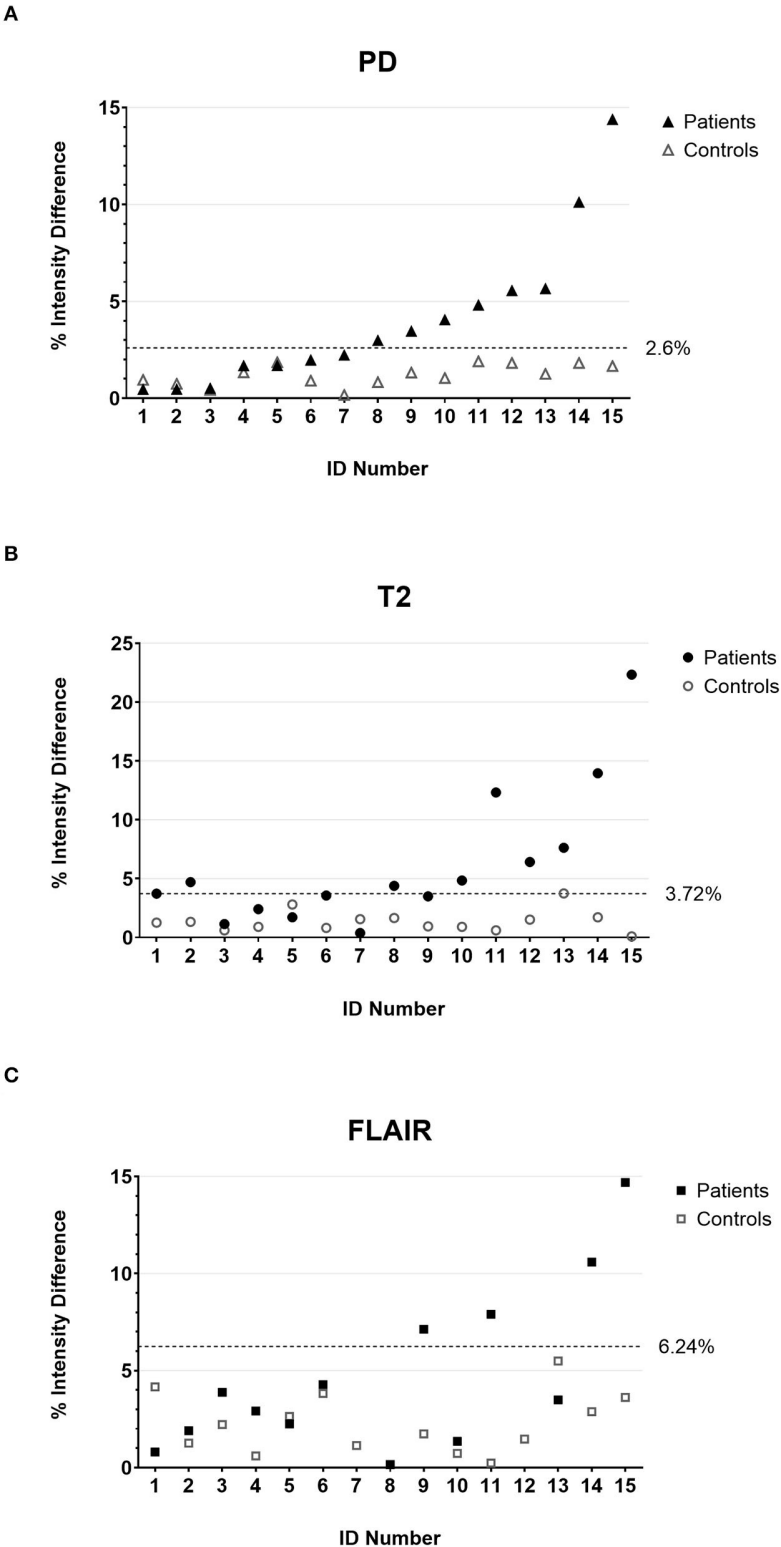
The percentage intensity differences between the two anterior quadrants of the medulla oblongata for all individual patients (filled marks) and controls (empty marks) calculated on PD-w **(A)**, T2-w **(B)**, and FLAIR **(C)**. The dotted lines indicate the threshold below that 99% of the value distribution of disease controls is to be expected. Accordingly, values above the threshold are deemed indicative of HOD in the patient cohort. It is noted that patient number 2 has a false-positive finding in T2-w **(B)** due to T2 hypointensities in the left anterior medulla oblongata. PD, proton-density; FLAIR, fluid-attenuated inversion recovery; HOD, hypertrophic olivary degeneration.

As a first step of the quantitative analysis, we calculated the mean percentage difference of the intensity values in the anterior quadrants of the medulla oblongata that contain the ION (Figure 1D) for the control cohort. The mean differences were comparable in the PD-w and T2-w datasets (1.21 and 1.37%, respectively; *p* = 0.55; paired *t*-test) while being significantly more pronounced in the FLAIR datasets (2.14%; PD-w vs. FLAIR *p* = 0.04/ T2-w vs. FLAIR *p* = 0.06; paired *t*-test) (Figure 2). Using these data to approximate the value distribution for healthy subjects, we calculated the 99% threshold for the intensity differences for PD-w (2.6%), T2-w (3.7%), and FLAIR (6.2%). Analyzing the data from the patient cohort, we found that 53.3% of the patients exceeded this threshold in the PD-w and T2-w datasets (Figures 2A,B), while only 30.8% of the patients exceeded the threshold in the FLAIR datasets (Figure 2C). It is noted that all but one patient exceeded the threshold in both PD-w and T2-w. The patient who only exceeded the threshold in PD-w showed an intensity difference close to the threshold in T2-w (3.5%; patient ID Number 9, Figure 2A, B). Conversely, there was one false-positive finding in the T2-w datasets with a higher intensity appearing contralateral to where a HOD was to be expected based on the causative index lesion (patient ID Number 2, Figure 2B).

**Figure 3:**
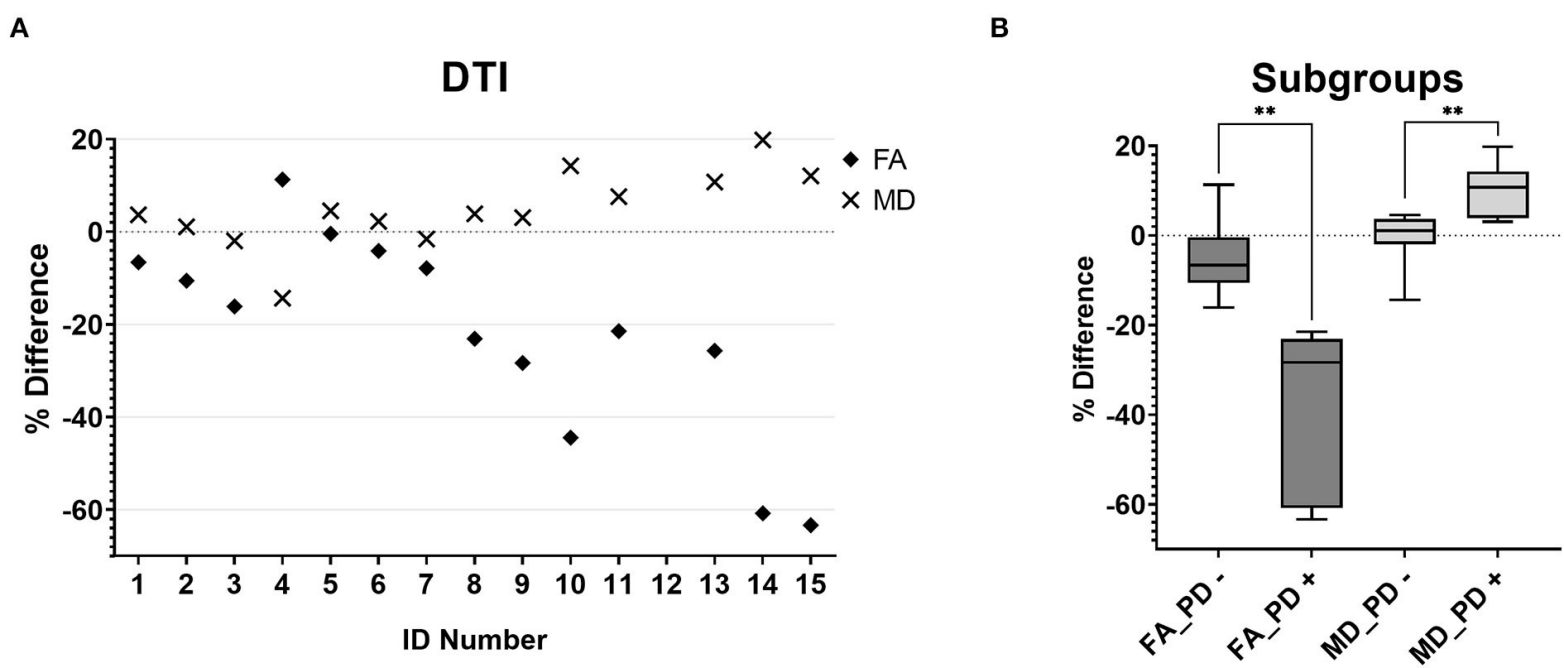
MD (diamonds) and FA (crosses) percentage differences between the two anterior quadrants of the medulla oblongata for all individual patients **(A)**. The boxplots [**(B)**, boxes extend from the 25th to 75th percentiles with a line indicating the median, whiskers indicate the minimum and maximum] demonstrate that patients who had conspicuous findings in the quantitative PD analysis showed significantly lower FA (FA_PD +, *p* = 0.006) and MD (MD_PD +, *p* = 0.002) values on the side where a HOD was to be expected than patients without conspicuous findings in the quantitative PD analysis (FA_PD-; MD_PD-). MD,mean diffusivity; FA, fractional anisotropy; PD, proton-density; HOD, hypertrophic olivary degeneration.

The MD and FA maps were only available for the patient cohort. The evaluation revealed a tendency toward lower FA and higher MD values on the side of the medulla oblongata where a HOD would be expected based on the location of the index lesion (Figure 3A). Considering the findings in the PD-w datasets, two subgroups were established, namely, patients with or without indication of HOD in the PD-w datasets (PD+ and PD-). Subsequently, in comparison with the PD- subgroup, the PD+ subgroup showed significantly higher MD and lower FA values on the side of the medulla oblongata where a HOD was to be expected [mean MD difference (-0.9 vs. 10.2%; *p* = 0.006)/mean FA difference (-4.9 vs. −38.2%; *p* = 0.002); Welch test; Figure 3B].

## Discussion

This prospective study aimed to assess the reliability of detecting HOD in the common rater-based approach on T2-w, FLAIR, and PD-w MRI, as well as alternative, quantitative analyses including DTI. As the main findings of the reviewer-based analysis, the interrater reliability was best for FLAIR, followed by T2-w and PD-w (Fleiss κ = 0.87 / 0.77 / 0.65), and HOD was more likely to be diagnosed based on PD-w than on T2 or FLAIR by the blinded raters. Importantly, however, false-positive findings of HOD were much less frequent in T2-w compared with PD-w and FLAIR. Quantitative analyses showed a significant increase in PD-w intensity differences between the left and right ION in 53.3% of patients with strokes in the GMT. Fittingly, these patients also showed significantly decreased FA and increased MD values in the medulla oblongata on the side of expected HOD occurrence. The implications of the findings for clinical and research practice will be outlined in the following.

The HOD is an MRI-based diagnosis relying on the visually detectable increase in signal intensity on T2-w/FLAIR sequences. The lack of objectivity in such an assessment is a limitation for future prospective studies. Even though epidemiologic data are missing, HOD is likely a rare disease that is not fully understood (23). Although there are reports of HOD without visible causative lesions on MRI (36), the correlation of HOD development with preceding lesions in the GMT is undoubtedly proven, and for a circumscribed lesion within the GMT, the side of HOD occurrence is well–predictable (2, 10). While the actual etiology of the underlying index lesion seems to be non-relevant (18, 26, 37, 38), our study focused on stroke patients, because this allowed for both a precise localization of the index lesion on MRI and a precise determination of the index lesion onset. Nevertheless, it is yet unknown how frequently a lesion within the GMT causes a HOD and to what extent the anatomical structures in the GMT must be affected. As we included patients prior to the diagnosis of HOD to avoid a sampling bias, it is reasonable to assume that not all recruited patients developed HOD. Together with the missing gold standard for a HOD diagnosis, this made it difficult to assess the actual sensitivity and specificity of the diagnostic approaches, while a comparison of different sequences and methods was feasible.

For the reviewer-based approach, we analyzed a standard FLAIR sequence and a double echo T2/PD-w sequence with thin slices and high in-plane resolution (voxel size 0.6 × 0.6 × 2 mm). While the healthy ION is not visible in both FLAIR and T2-w, it can be identified as a hyperintense structure to some degree in the PD-w images (Figure 2, refer to Supplementary Figure 1 for additional examples). To allow the reviewers to adapt to the appearance of the normal ION in the PD-w sequence, four test datasets were handed out before the actual review. As hypothesized with regard to former studies (28, 39), the PD-w sequence had the highest sensitivity based on the number of HOD diagnoses. Yet, it also had the lowest interrater reliability and the highest number of false-positive findings (Table 2). This suggests that the employed PD-w sequence is sensitive, yet a distinct separation of normal and pathological findings seems to prove difficult. The sensitivity of both T2-w and FLAIR was comparable but there was a higher rate of false-positive findings on FLAIR images. This might be attributable to a higher susceptibility of the FLAIR sequence to artifacts (40), particularly in the brain stem, and seems to be reflected in the higher intensity differences between the left and right side of the medulla oblongata in disease controls compared with T2-w (Figures 2B,C). Overall, our data suggest that the use of a T2-w sequence is most suitable for a rater-based approach and is likely very robust in relation to specificity. Concerning retrospective studies analyzing MRI data that have not been optimized for the imaging of the brain stem, the question remains to what degree the validity of a reviewer-based approach can be affected by the imaging quality and resolution.

For our quantitative analysis, we refrained from a direct segmentation of the ION as described in previous studies (18, 21, 41). The normal ION is not identifiable on T2-w and FLAIR images (21) and often displays a diffuse T2 hyperintensity if a HOD is present. Consequently, the reproducibility of a direct segmentation is questionable, especially for evaluation in clinical routine. Therefore, we used a segmentation of the two anterior quadrants of the medulla oblongata, relying only on easily identifiable anatomic landmarks (anterior median fissure and posterolateral sulcus; Figure 1D). We chose the craniocaudal ROI dimension of 8 mm as it covered most of the medulla oblongata in all 30 subjects while preventing the inclusion of adjacent structures. The ensuing ROIs were relatively large in size and thereby robust but included not only the ION but also the pyramidal tracts. Since we normalized our measurements in individual subjects to the contralateral side, the partial volume effect should nevertheless be largely suppressed in the final result.

The inclusion of an age- and sex-matched control group allowed for a valid approximation of the signal differences of the left- and right-sided ROI in a healthy population. Even though we set the threshold for the assumption of a pathological finding higher than the usually accepted significance level (α <0.01 rather than 0.05), 53.3% of patients showed conspicuous findings in the analysis of the PD-w datasets compared with the control group (Figure 2A). The result was almost the same for the analysis of the T2-w datasets (Figure 2B). As a major difference, there was one false-positive finding in the T2-w datasets that, upon review, was attributable to a hypointensity in one ROI rather than a hyperintensity in the other. In contrast, the evaluation of the FLAIR datasets yielded a lower rate of 30.8% of patients exceeding the calculated threshold. A possible explanation for this deviation is the higher spread of the FLAIR data (Figure 2C), which could be attributable to susceptibility to artifacts. When including the analysis of the FA and MD maps, we found significantly higher differences between the left- and right-sided ROI in patients who also had significant findings in the analysis of the PD-w datasets (Figure 3). Distinctly lower FA values suggested a decrease in the directionality of the diffusion, which can be regarded as a surrogate for demyelination and the loss of neuronal fibers (42). In addition, the increase of the MD implies a general increase in diffusion and can be explained through a decrease in cell density, either caused by cell death or extracellular edema (42). Both findings are largely in line with previous DTI studies of HOD (21, 29, 31) and correspond well to the histopathological findings in HOD (9, 10). This supports the prior assumption that high differences of PD-w intensity in the left and right anterior quadrant of the medulla oblongata indeed indicate the presence of HOD. In comparison with the rater-based approach, the sensitivity of the quantitative analysis based on the PD-w datasets might be higher, especially if unanimous ratings are required (53.3% vs. 40% HOD diagnosis).

In summary, our study suggests the use of T2-w images for the rater-based diagnosis of HOD rather than FLAIR or PD-weighted sequences. Especially concerning the specificity, the rater-based approach yielded good results. As an alternative, a reproducible, quantitative HOD diagnosis based on PD-w and possibly additional DTI data seems feasible and might have a higher sensitivity than the rater-based alternative. Especially for future prospective studies, it might be of interest to include quantitative diagnostic measures to improve the repeatability and comparability of the results.

### Limitations

First, the small size of our patient cohort limits the power of the statistical analysis. Therefore, further studies are needed to prove that our findings are generalizable. Yet, due to the rarity of the disease, larger, prospective patient collectives do not exist (20, 21), and our study could serve as a starting point for future analyses. Concerning our quantitative analysis, the inclusion of an age- and sex-matched control cohort allowed for a substantial point of reference for the evaluation of our results but an external validation in an independent patient cohort is still needed and will be addressed in the future. The unavailability of two FLAIR datasets in the patient cohort poses a risk for bias, especially in the reviewer-based analysis; yet, the assumption that FLAIR might be less reliable than T2 is supported by the quantitative analysis of the 15 disease controls. Lastly, our study did not consider the possibility of a bilateral HOD. A bilateral involvement of the ION is known to appear if bilateral lesions in the GMT are present but might also occur after a unilateral injury or in non-lesional, idiopathic HOD (36). Nonetheless, in our experience, bilateral HOD after unilateral injuries is an asymmetric finding with a marked emphasis on the expected side and is likely present within our dataset (Supplementary Figure 1). As it is most important for future studies on the epidemiologic symptoms and therapy of HOD to reliably assess the presence of a HOD, our study did focus on establishing the diagnosis rather than the extent.

## Data availability statement

The raw data supporting the conclusions of this article will be made available by the authors upon reasonable request.

## Ethics statement

The studies involving human participants were reviewed and approved by the Review Board of the Ethical Committee at the University Hospital Frankfurt as of February 10th, 2020, without further requests (project number: 19-467). The patients/participants provided their written informed consent to participate in this study.

## Author contributions

Conceptualization: CF, ES, MS-P, EH, and MS. Formal analysis and investigation: ES, MS-P, MR, SB, JS, MG, and MS. Writing (original draft preparation): ES and MS-P. Writing (review and editing): all authors. Resources: EH and CF. Supervision: EH and CF. All authors contributed to the article and approved the submitted version.

## Funding

This study was being funded by the junior researcher scholarship program of the Faculty of Medicine at the Goethe University Frankfurt.

## Conflict of interest

The authors declare that the research was conducted in the absence of any commercial or financial relationships that could be construed as a potential conflict of interest.

## Publisher's note

All claims expressed in this article are solely those of the authors and do not necessarily represent those of their affiliated organizations, or those of the publisher, the editors and the reviewers. Any product that may be evaluated in this article, or claim that may be made by its manufacturer, is not guaranteed or endorsed by the publisher.
